# Association Between Serum Follicle-Stimulating Hormone and Sarcopenia and Physical Disability Among Older Chinese Men: Evidence From a Cross-Sectional Study

**DOI:** 10.3389/fmed.2021.724649

**Published:** 2022-01-04

**Authors:** Yingying Ke, Jun Xu, Xiaoyan Zhang, Qihao Guo, Yunxia Zhu

**Affiliations:** Department of Geriatrics, Shanghai Jiao Tong University Affiliated Sixth People's Hospital, Shanghai, China

**Keywords:** follicle-stimulating hormone, sarcopenia, physical performance, disability, older men

## Abstract

**Background:** Sarcopenia is a geriatric syndrome characterized by progressive loss of muscle mass, function and quality and associated with a range of adverse health outcomes including disability. Despite a negative correlation between muscle mass and follicle-stimulating hormone (FSH) levels in postmenopausal women, it is unclear if FSH is associated with sarcopenia and its poor outcomes, especially in older men.

**Methods:** We used cross-sectional data from 360 men aged over 80 who participated in health check-ups to investigate correlations between serum FSH and sarcopenia, individual sarcopenia components, low physical performance (gait speed ≤ 0.8 m/s) and instrumental activities of daily living (IADL) disability. Sarcopenia and severe sarcopenia were diagnosed according to the revised definition of the European Working Group on Sarcopenia in Old People (EWGSOP2).

**Results:** The prevalence of sarcopenia was 17.8% in this population. In binary logistic regression analysis, compared with higher FSH group, lower FSH group showed a significant reduction in the risk of low calf circumference (a surrogate for muscle mass; OR 0.308, 95% CI 0.109–0.868, *P* = 0.026) after adjusting potential confounders including age, waist circumference, education, exercise, associated biochemical parameters, other sex hormones and high-sensitivity C-reactive protein. The correlation between FSH and low handgrip strength was marginally significant (OR 0.390, 95% CI 0.151–1.005, *P* = 0.051). No associations were observed between FSH and sarcopenia, severe sarcopenia, and disability in adjusted models.

**Conclusion:** In older men, circulating FSH was not associated with sarcopenia, sarcopenia severity, the majority of its components and adverse health outcome (IADL disability), with the exception of low calf circumference. Further work is needed to better elucidate the association of FSH and low muscle quantity by adopting more accurate measurement method of appendicular skeletal muscle mass such as DXA, CT or MRI.

## Introduction

Sarcopenia has been defined as a geriatric syndrome characterized by progressive loss of muscle mass, muscle strength and physical performance that is associated with a range of adverse health outcomes including frailty, disability, falls and mortality (1). In addition to common risk factors such as advanced age, inactivity and poor nutritional condition (1), reproductive hormones, which also change with aging (2), have been associated with sarcopenia (3). Among reproductive hormones, the effects of androgens and estrogens on skeletal muscle anabolism and homeostasis are well-established, particularly combined with resistance exercise training (4, 5), although caution should be taken regarding side-effects when sex steroids supplementation is applied in clinical practice (6).

Progress and updates have been made in the association of reproductive hormones and sarcopenia since extragonadal actions of follicle-stimulating hormone (FSH) were described in animals and humans. FSH has the potential to induce lipid storage, redistribution, and ectopic deposition (7–9), all factors that have been linked to chronic low-grade inflammatory state which has been recognized as a cause of age-related sarcopenia (10). For example, high-sensitivity C-reactive protein (hs-CRP), a biomarker of systemic inflammation, was found to be independently associated with sarcopenia component in a recent meta-analysis (11). Accordingly, the potential regulation of FSH on skeletal muscle mass has been proposed in the past decade, in which lean mass (mainly muscle) is negatively correlated with FSH levels in both young and old postmenopausal women (12–15). Most recently, observations from Park et al. that a reduction in appendicular lean mass across menopausal stages was associated with higher FSH levels supplied further evidence for the potential unfavorable effect of FSH on skeletal mass loss (16). However, these studies were all conducted in postmenopausal or perimenopausal women as rapid elevation of FSH level during perimenopausal transition and remain high after menopause. Research focusing on older males, a subpopulation with a high prevalence of sarcopenia and associated adverse outcomes such as disability, is still insufficient, although a gradual and constant increase in circulating FSH also exists in men as aging (2) and the upregulation of FSH in males receiving androgen deprivation therapy promotes the development of several metabolic diseases such as metabolic syndrome, atherosclerotic cardiovascular disease, and insulin resistance (17). At present, current research has not investigated the link between FSH and muscle strength, a key component for defining sarcopenia, in both males and females. Thus, there is a need to investigate the effects of FSH in sarcopenia and associated muscle function in older individuals, especially in older males.

In this cross-sectional study based on males aged over 80 years old, we first determined the level of FSH in individuals with or without sarcopenia. Next, we assessed the associations between serum FSH level and sarcopenia, physical performance and disability after controlling for potential confounders including age, obesity, exercise, education level, lipid profiles, nutrition status, systemic inflammation, and other hormones.

## Materials and Methods

### Study Design

The cross-sectional baseline data used in the present study were from a single-center, prospective observational study which initiated to assess FSH, metabolic risks, and aging in elderly subjects underwent annual health examination in the Department of Geriatrics of the Shanghai Jiaotong University Affiliated Sixth People's Hospital (ChiCTR1800018015; www.chictr.org.cn). The baseline data were collected from January 2019 to December 2019.

### Eligibility Criteria and Sample Selection

**Figure 1:**
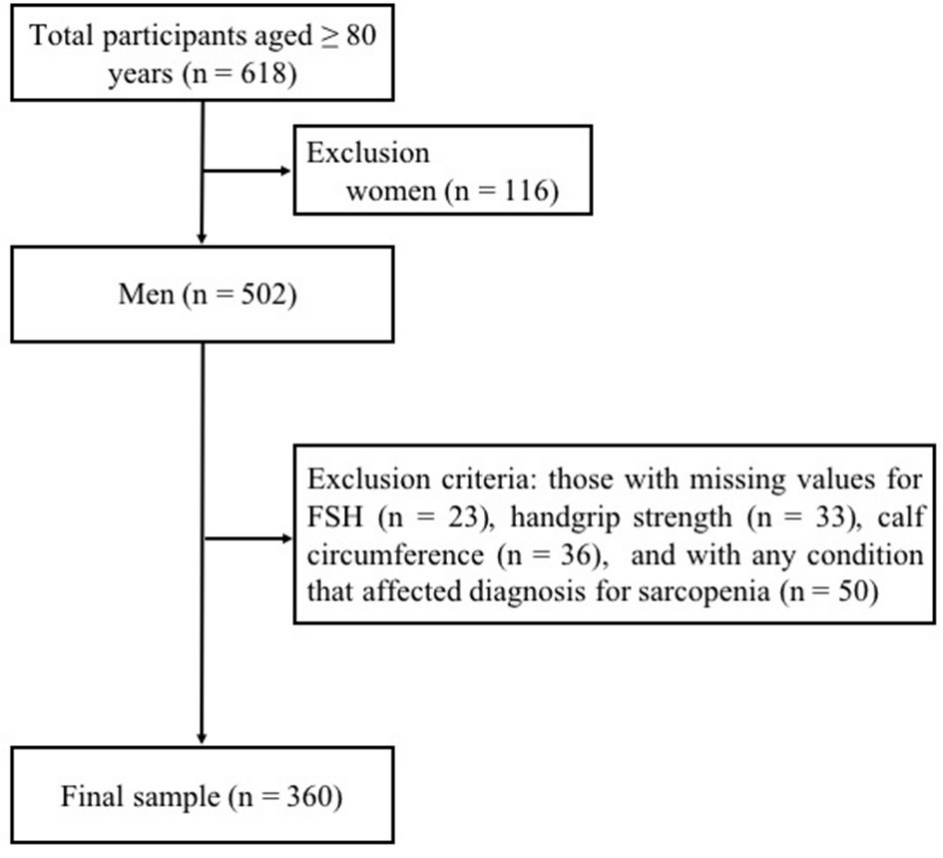
Flowchart for participants enrolled in this study.

The recruitment criteria of the original prospective study were as follows: (i) 80 years of age or older; (ii) no problems of communication; (iii) no acute or end-stage illness; (iv) without taking any sex hormonal replacement therapy or with diseases involving in the hypothalamo-pituitary-gonadal/thyroid/adrenal axis. At baseline, 618 volunteers, of which 502 were men and 116 were women, were recruited *via* advertisement in the hospital. In order to investigate the cross-sectional association of FSH and sarcopenia in old men in the present analysis, subjects who had the following conditions at baseline were excluded from the study: (i) those with missing values for FSH (*n* = 23), handgrip strength (*n* = 33) or calf circumference (*n* = 36); (ii) females (*n* = 116); (iii) with any condition that affected detection for handgrip strength and calf circumferences, such as upper limb arthritis and edema of lower limbs (*n* = 50). Finally, 360 males aged 80–98 years were included in the study (Figure 1).

### Outcome Measures

Data collection of all variables was organized by the principal investigator and was performed by a well-trained research team. Data on demographics, smoking, exercise, comorbidities, anthropometric and circulating biochemical measurements were collected. Among the above indicators, only those which had shown statistical differences (*P* < 0.05) between sarcopenia sub-groups in the comparison analysis were treated as covariates in the subsequent binary logistic regression analysis. As such, age, waist circumference, education, exercise, luteinizing hormone (LH), estradiol (E_2_), total testosterone (TT), triglyceride (TG), low-density lipoprotein cholesterol (LDL-C), 25-hydroxyvitamin D [25(OH)D], albumin, free triiodothyronine (FT3) and hsCRP were selected as co-variables, all introduced in the adjusted regression models.

#### Assessment of Sarcopenia

For the diagnosis of sarcopenia, calf circumference, handgrip strength and 4-meter gait speed were assessed. Calf circumference was measured with the elderly participant in standing position, at the greatest circumference of the lower right leg, recorded in centimeters. The handgrip strength of the dominant hand was the maximum value evaluated three times using CAMRY hydraulic hand dynamometer (EH101; Camry, China). At least 1-min rest was required between each test. 4-meter gait speed test is performed twice to obtain the gait speed in which the participants walk 4 meters at their usual pace. The gait speed was calculated by dividing the distance (meter) by walking time (second). The European Working Group on Sarcopenia in Older People updated the consensus definition of sarcopenia in 2018 (EWGSOP2) (18) as the following criteria: (1) low muscle strength, (2) low muscle quantity or quality and (3) low physical performance. Probable sarcopenia is identified by criterion one, while the diagnosis is confirmed by additional documentation of criterion two. Severe sarcopenia is considered if all criteria are met. In the present study, handgrip strength <27 kg was considered low handgrip strength and calf circumference <31 cm was considered low muscle quantity (low calf circumference), according to EWGSOP2 recommended cutoffs. Low physical performance was defined as a 4-meter gait speed ≤ 0.8 m/s.

#### Assessment of Disability

Physical disability was assessed by instrumental activities of daily living (IADL) (19, 20). In brief, the IADL index includes eight tasks—shopping, preparing meals, using the telephone, housekeeping, laundry, transportation, taking medications, and managing money. Each IADL item was scored 0 to 1 according to the ability of participants to perform each task and total score was eight. Scores ranged from 0 to 8 with a lower score denoting the need for assistance with IADLs. A score ≤ 7 was defined as IADL disability.

#### Laboratory Measurements

After an overnight fast, venous blood samples were collected from all individuals and the samples were used to measure fasting blood glucose (FPG), hemoglobin A1c (HbA1c), TG, total cholesterol (TC), LDL-C, high-density lipoprotein cholesterol (HDL-C), hsCRP, albumin, 25(OH)D and hormones. Plasma fasting glucose was measured using the glucose oxidase method (detection range: 0.078–55.51 mmol/L). HbA1c was measured by high-performance liquid chromatography (Bio-Rad Laboratories) (detection range: 3.5–20%). Serum lipids were measured by standard enzymatic methods using a Hitachi 747 analyzer (Castle Hill) (detection range of TC: 0.05–12.90 mmol/L, TG: 0.01–22.58 mmol/L, LDL-C:0.05–10.20 mmol/L, HDL-C: 0.10–2.59 mmol/L). hsCRP was detected by particle-enhanced immunoturbidimetry (Dade Behring Inc.) (detection range: 0.175–500 mg/L). Serum albumin was measured by the bromocresol green method (Shanghai Kehua Bio-Engineering Co, Ltd) (detection range: 10.0–60.0 g/L). Hormones including serum 25(OH)D (detection range: 3.0–70.0 ng/ml), FT3 (detection range: 0.4–50.0 pmol/L), free thyroxine (FT4, detection range: 0.3–100.0 pmol/L) and thyroid-stimulating hormone (TSH, detection range: 0.06–99 mIU/L) were quantified by an electrochemiluminescence immunoassay method (Roche Diagnostics GmbH) on a Cobas e601 analyzer.

Reproductive hormones TT (detection range: 0.087–52.000 nmol/L), E_2_ (detection range: 18.35–15781.00 pmol/L), FSH (detection range: 0.5–160.0 IU/L), and LH (detection range: 0.1–200.0 IU/L) were detected by chemiluminescence (Abbott GmbH & Co. KG) with inter- and intra-assay coefficients of variations of <5%. The normal range was 6.68–25.7 nM for TT, 41.4–159 pM for E_2_, 1.5–12.4 IU/L for FSH, and 1.7–8.6 IU/L for LH.

#### Other Covariates

Waist circumference was measured at the middle point between the lower edge of the rib cage and iliac crests. Education was dichotomized with a cutoff for college graduation vs. no college graduation due to the high proportion of subjects with college graduation (80.6 %). Subjects with at least a college graduation were regarded as having high education. Living alone was defined based on living without a partner. We designated routine exercises with moderate to strenuous intensity, three times a week or more frequently. Non-smoker were defined as those who had never smoked or had smoked fewer than 100 cigarettes in the past. Comorbidities included type two diabetes (T2DM), coronary heart disease (CHD), hypertension, chronic obstructive pulmonary disease (COPD), arrhythmia, chronic kidney disease (CKD), osteoporosis, history of fracture, osteoarthritis, and neoplasms. And the number of comorbidities in every participant was calculated.

### Statistical Analysis

Data were tested for normality using the Shapiro-Wilk test, and continuous variables are presented as mean ± standard deviation for normally distributed variables or median (25th percentile to 75th percentile) for skewed variables, whereas categorical variables are expressed as numbers with percentages. Differences between two groups (non-sarcopenia and sarcopenia) were compared by the Student's *t*-test for data with a normal distribution or the Mann-Whitney U test for data with a skewed distribution. For categorical variables, intergroup comparisons were analyzed using the chi-square test. The association between FSH (categorical variables) and sarcopenia, severe sarcopenia, two components for sarcopenia diagnosis, lower physical performance, and IADL disability was assessed using a binary logistic regression model, organized in crude and adjusted models, and results are expressed as odds ratios (OR) with a 95% confidence interval (CI). All statistical analyses were performed using SPSS 26.0 (SPSS Inc., Chicago, IL). A two-sided *P* < 0.05 was considered statistically significant.

## Results

Overall, we enrolled 360 old men (with a mean age of 86.2 ± 4.4 years). The mean number of comorbidities was 3.8 ± 1.7 per volunteer. Of the total number of participants, 64 (17.8%) had sarcopenia according to the EWGSOP2 definition. Table 1 summarized the general demographic and laboratory characteristics of these subjects according to their sarcopenia status. As expected, those with sarcopenia had a higher hsCRP but a lower 25(OH)D (both *P* < 0.05), FT3 and proportion of high education (both *P* < 0.01), than those without sarcopenia. In addition, sarcopenic individuals had a decreased proportion of routine exercises when compared to those of non-sarcopenic individuals (*P* < 0.01). Poorer nutritional status indicated as lower TG, LDL-C (both *P* < 0.05), albumin and waist circumference (both *P* < 0.01) was also detected in sarcopenic participants compared to non-sarcopenic ones. Moreover, men with sarcopenia were older (*P* < 0.01), coupled with higher circulating FSH and LH levels (both *P* < 0.05), whereas no differences in TT and E_2_ were found between the two groups.

**Table 1:** Characteristics of study population according to sarcopenia status.

**Variable**	**non-sarcopenia** **(*n* = 296)**	**Sarcopenia** **(*n* = 64)**	***P***
Age (years)	85.8 ± 4.0	88.1 ± 4.3	<0.001
Waist circumference (cm)	91.2 ± 9.3	86.2 ± 8.9	<0.001
Non-smoker (%, *n*)	74.3 (220)	81.3 (52)	0.242
High education (%, *n*)	84.8 (251)	67.2 (43)	0.001
Living alone (%, *n*)	8.8 (26)	7.8 (5)	0.802
Routine exercise (%, *n*)	55.1 (163)	26.6 (17)	<0.001
Number of Comorbities	3.7 ± 1.6	4.0 ± 1.8	0.120
FPG (mmol/L)	5.1 (4.7–5.7)	5.0 (4.7–5.4)	0.387
HbA1c (%)	6.2 ± 0.9	6.4 ± 1.2	0.145
TC (mmo/L)	4.1 ± 1.0	3.8 ± 0.9	0.071
TG (mmo/L)	1.1 ± 0.7	0.9 ± 0.5	0.038
HDL-C (mmo/L)	1.2 ± 0.3	1.3 ± 0.4	0.111
LDL-C (mmo/L)	2.2 ± 0.8	2.0 ± 0.7	0.036
25(OH)D (ng/mL)	16.6 (12.2–24.5)	16.0 (9.9–19.0)	0.038
Albumin (g/L)	40.2 ± 3.4	37.8 ± 4.7	<0.001
hsCRP (mg/L)	1.00 (0.40–2.44)	1.53 (0.82–4.52)	0.024
FT3 (pmol/L)	4.1 ± 0.6	3.7 ± 0.7	<0.001
FT4 (pmol/L)	16.4 ± 2.3	16.3 ± 1.7	0.702
TSH (mIU/L)	3.2 ± 2.5	3.3 ± 3.0	0.968
TT (nmol/L)	13.0 ± 6.3	14.3 ± 7.7	0.137
E_2_ (pmol/L)	130 ± 46	142 ± 49	0.061
FSH (IU/L)	18.7 (12.1–38.3)	29.5 (14.8–37.0)	0.038
LH (IU/L)	9.4 (6.8–19.0)	12.5 (9.6–23.9)	0.033

*Data are presented as means ± standard deviation (SD) for data with a normal distribution, medians (interquartile range) for data with a skewed distribution, or as a number with a proportion for categorical variables. The differences between non-sarcopenia and sarcopenia were compared by the Student's t-test for data with a normal distribution or the Mann-Whitney U test for data with a skewed distribution. For categorical variables, differences were analyzed using the chi-square test*.

*FPG, fasting blood glucose; HbA1c, hemoglobin A1c; TC, serum total cholesterol; TG, triglyceride; HDL-C, high-density lipoprotein cholesterol; LDL-C, low-density lipoprotein cholesterol; 25(OH)D, 25-hydroxyvitamin D; hsCRP, high-sensitivity C-reactive protein; FT3, free triiodothyronine; FT4, free thyroxine; TSH, thyroid-stimulating hormone; TT, total testosterone; E_2_, estradiol; FSH, follicle-stimulating hormone; LH, luteinizing hormone*.

We next stratified participants into two groups: lower FSH and higher FSH groups, according to the median FSH value measured in our assays (20.42 IU/L). As shown in Table 2, compared with the lower FSH group, men with a higher FSH had decreased handgrip strength and calf circumference (both *P* < 0.05). There was no difference in 4-meter gait speed between two groups.

**Table 2:** Sarcopenia associated components in older males stratified by the follicle-stimulating hormone (FSH) median value.

**Variable**	**Lower-FSH** **(*n* = 181)**	**Higher-FSH** **(*n* = 179)**	***P***
Handgrip strength (kg)	22.9 ± 7.0	21.3 ± 6.5	0.029
Calf circumference (cm)	32.8 ± 3.0	31.6 ± 4.0	0.020
Gait speed (m/s)	0.78 ± 0.31	0.73 ± 0.27	0.111

*Data are presented as means ± standard deviation (SD)*.

Table 3 shows the association of FSH with sarcopenia, its defining components and measures of physical function in aged men analyzed by multinomial logistic regressions. We calculated the ORs in models with and without adjustment for potential confounders. In the crude analysis, higher FSH group had increased ORs for sarcopenia (0.575, 95% CI 0.331–0.999, *P* = 0.049), low calf circumference (0.522, 95% CI 0.307–0.887, *P* = 0.016), and low handgrip strength (0.386, 95% CI 0.235–0.636, *P* < 0.001). Adjusted Model was adjusted for age, waist circumference, education, exercise, LH, E_2_, TT, TG, LDL-C, 25(OH)D, albumin, FT3, and hsCRP. There were no significant associations observed between FSH and sarcopenia (OR 0.511, 95% CI 0.178–1.467, *P* = 0.212) or severe sarcopenia (OR 0.400, 95% CI 0.136–1.175, *P* = 0.096) after controlling above confounders, although the prevalence of sarcopenia was significant different between two groups (*P* < 0.05). For sarcopenia components, lower FSH men had decreased ORs for low calf circumference (OR 0.308, 95% CI 0.109–0.868, *P* = 0.026) compared with higher FSH men. The correlation between FSH and low handgrip strength was marginally significant (OR 0.390, 95% CI 0.151–1.005, *P* = 0.051). No significant association was observed between FSH and low physical performance between two groups (*P* = 0.446).

**Table 3:** Multivariate-adjusted logistic regression analyses of associations between follicle-stimulating hormone (FSH) levels and sarcopenia, severe sarcopenia, and individual sarcopenia components.

	**Prevalence** **(%, *n*)**	**Crude model**	***P***	**Adjusted model^a^**	***P***
**Sarcopenia**					
Higher-FSH	21.8 (39)^*^	1.00		1.00	
Lower-FSH	13.8 (25)	0.575 (0.331–0.999)	0.049	0.511 (0.178, 1.467)	0.212
**Severe sarcopenia**					
Higher-FSH	19.6 (35)	1.00		1.00	
Lower-FSH	12.7 (23)	0.599 (0.338–1.062)	0.079	0.400 (0.136–1.175)	0.096
**Low calf circumference**					
Higher-FSH	25.1 (45)^*^	1.00		1.00	
Lower-FSH	14.9 (27)	0.522 (0.307–0.887)	0.016	0.308 (0.109, 0.868)	0.026
**Low handgrip strength**					
Higher-FSH	84.4 (151)^**^	1.00		1.00	
Lower-FSH	65.7 (119)	0.386 (0.235–0.636)	<0.001	0.390 (0.151,1.005)	0.051
**Low physical performance**					
Higher-FSH	59.8 (108)	1.00		1.00	
Lower-FSH	58.0 (105)	0.930 (0.611–1.415)	0.734	0.717 (0.305,1.685)	0.446

*Data were a proportion with a number for categorical variables and odds ratios (95% confidence interval). Low calf circumference: calf circumference <31 cm; Low handgrip strength: handgrip strength <27 kg; Low physical performance: 4-meter gait speed ≤ 0.8 m/s. All models constructed by logistic regression analysis: a: adjusted for age, waist circumference, education, exercise, LH, E_2_, TT, TG, LDL-C, 25(OH)D, albumin, FT3, and hsCRP*.

* *P < 0.05,*

** *P < 0.01, Lower-FSH group vs. Higher-FSH group*.

As sarcopenia was found to be an independent risk factor for several adverse health outcomes including disability (21), we next assessed the association between physical disability defined as an IADL score ≤ 7 and FSH levels (Table 4). No relationship of FSH and disability was observed in adjusted model although the significant association was observed in the crude model (*P* < 0.001) and the apparent difference in the proportion of IADL disability between two groups (*P* < 0.01).

**Table 4:** Multivariate-adjusted logistic regression analyses of associations between follicle-stimulating hormone (FSH) levels and IADL disability.

	**Prevalence (%, *n*)**	**Crude model**	**P**	**Adjusted model^a^**	***P***
**IADL disability**					
Higher-FSH	67.6 (121)^**^	1.00		1.00	
Lower-FSH	49.2 (89)	0.464 (0.302-0.711)	<0.001	0.664 (0.300, 1.467)	0.311

*Data were a proportion with a number for IADL disability and odds ratio (95% confidence interval). IADL disability: score ≤ 7. All models constructed by logistic regression analysis: a: adjusted for age, waist circumference, education, exercise, LH, E_2_, TT, TG, LDL-C, 25(OH)D, albumin, FT3, and hsCRP*.

** *P < 0.01, Lower-FSH group vs. Higher-FSH group*.

## Discussion

Here, we provide a comprehensive examination of the cross-sectional associations between FSH levels and sarcopenia and associated poor outcomes in men over 80 years of age after adjusting for potential confounders of clinical significance. To our knowledge, although there are a limited number of studies showing associations between FSH and muscle mass in females (12–15), there is currently no human study that has explored the relationship between FSH and sarcopenia in men. Hence, this is the first study that has assessed the link between serum FSH and sarcopenia and sarcopenia-associated functional outcomes including physical disability in males. After adjusting for potential variables involved in sarcopenia, we found that higher FSH concentrations may correlate with reduced skeletal muscle mass indicated as low calf circumference, but not sarcopenia, sarcopenia severity, low muscle strength and IADL disability.

Our study is consistent with previous observational studies conducted in young and old postmenopausal females, in which high FSH levels were associated with lean (mainly muscle) mass (12–15, 22). In men, there is only one study (AGES-Reykjavik study) that has examined this association, showing no correlation (14). There are three possible explanations for differences between the AGES-Reykjavik study and ours. One possibility is that our older Chinese men were exposed to higher circulating concentrations of FSH than that of older men in Iceland (mean FSH: 28 IU/L vs. 19 IU/L, both detected by ELISA), which could surpass the threshold level of FSH to affect muscle mass. Furthermore, we adjusted for potential covariates including age, waist circumference, education, exercise, LH, E_2_, TT, TG, LDL-C, 25(OH)D, albumin, FT3, and hsCRP, while the AGES-Reykjavik study only adjusted for age, subgroup, E_2_, and TT. Hence, the results of our study were more rigorous than those of the AGES-Reykjavik study. Third, the number of analytic samples in the present study (*n* = 360) is larger than those of the AGES-Reykjavik study (*n* = 245), which could lead to higher statistical power.

We found a possible association between skeletal muscle mass and FSH, but not between muscle strength and FSH after adjusting for all the covariates although muscle quantity is the basis for muscle strength. This suggests that the effect of FSH on muscle strength is small. In fact, other reproductive hormones such as androgens also presented a similar characteristic of inducing a greater promotion in muscle mass than muscle strength (23, 24). Thus, hormonal regulation of FSH in association with resistance exercise training may be a promising new strategy to manage sarcopenia since exercise remains a valid countermeasure against muscle atrophy and androgen treatment is not recommended due to intolerable adverse events in older men (6, 25). Notably, the genotype of FSH receptors may affect the role of circulating FSH on target tissues. A recent study reported that men with the GG genotype of the FSH receptor rs6166 SNP have lower levels of blood glucose than those with the AA genotype and their FSH concentrations were inversely correlated with insulin and insulin resistance. Meanwhile, the FSH receptor rs6166 A/G genotype did not affect glucose metabolism in healthy men (26). Thus, genotype-specific effects of FSH receptors on muscle mass, muscle strength and function should be explored in the future study.

Sarcopenia is associated with an increased likelihood of several adverse health outcomes including disability (21). We did not observe any association between FSH and gait speed or disability in the present study. A prospective study of Japanese community-dwelling older adults found that there were no increased risks of incident disability for participants with only one sarcopenia component (21). Another prospective study with 9.1 years follow-up revealed that gait speed was a powerful predictor of disability, in men, compared with sarcopenia components (27). Thus, it is reasonable for the loss of association between FSH and gait speed and disability in our study. In addition, functional disability in older men has multiple causes, of which muscle weakness is only one. Other risk factors such as dietary pattern and anemia, may also play important roles in the presence of disability among older adults, which were not analyzed in the present study (28, 29).

We acknowledge some limitations to the present study. First, although a possible association between FSH and skeletal muscle mass was observed, as a cross-sectional study, the causality of this relationship cannot be established. Second, in the current study, calf circumference was not as accurate as bioelectrical impedance analysis or dual-energy X-ray absorptiometry for evaluating muscle mass especially in individuals with a high percentage of body fat, as it can equally predict lean and fat mass. However, calf circumference, as a validated surrogate measure of skeletal muscle mass, was suggested by EWGSOP2 as it is easy to perform in clinical and population settings. Finally, the participants were from a population receiving regular medical examination as opposed to a community or a general population, so our results might not be generalizable.

## Conclusion

In our cross-sectional study of elderly Chinese men aged over 80, FSH was not associated with sarcopenia, sarcopenia severity, the majority of its components and sarcopenia-associated adverse outcome (IADL disability). Interestingly, FSH seems to be a independent risk factor for low skeletal muscle mass indicated as low calf circumference although calf circumference is not a gold standard method to evaluate muscle mass. Further study is needed to confirm the association of FSH and low muscle quantity by adopting more accurate measurement method of appendicular skeletal muscle mass such as DXA, CT or MRI.

## Data Availability Statement

The original contributions presented in the study are included in the article/supplementary material, further inquiries can be directed to the corresponding author.

## Ethics Statement

The studies involving human participants were reviewed and approved by the Ethics Committee of Shanghai Jiaotong University Affiliated Sixth People's Hospital (Reference number: 2018–109). The patients/participants provided their written informed consent to participate in this study.

## Author Contributions

YK and YZ: designed the study. YK, XZ, and YZ: collected and analyzed the data. YK: wrote the manuscript. QG and YZ: participated in the critical review of the manuscript. All authors read and approved the final manuscript.

## Funding

The financial support for this study comes from Clinical Research Fund of Shanghai Sixth People's Hospital (ynlc201828).

## Conflict of Interest

The authors declare that the research was conducted in the absence of any commercial or financial relationships that could be construed as a potential conflict of interest.

## Publisher's Note

All claims expressed in this article are solely those of the authors and do not necessarily represent those of their affiliated organizations, or those of the publisher, the editors and the reviewers. Any product that may be evaluated in this article, or claim that may be made by its manufacturer, is not guaranteed or endorsed by the publisher.
